# EMQIT: a machine learning approach for energy based PWM matrix quality improvement

**DOI:** 10.1186/s13062-017-0189-y

**Published:** 2017-08-01

**Authors:** Karolina Smolinska, Marcin Pacholczyk

**Affiliations:** Institute of Automatic Control, Silesian University of Technology, Akademicka 16, 44-100 Gliwice, Poland

**Keywords:** PWM matrix, TRANSFAC, Jaspar, TFBS, 3DTF

## Abstract

**Background:**

Transcription factor binding affinities to DNA play a key role for the gene regulation. Learning the specificity of the mechanisms of binding TFs to DNA is important both to experimentalists and theoreticians. With the development of high-throughput methods such as, e.g., ChiP-seq the need to provide unbiased models of binding events has been made apparent. We present EMQIT a modification to the approach introduced by Alamanova et al. and later implemented as 3DTF server. We observed that tuning of Boltzmann factor weights, used for conversion of calculated energies to nucleotide probabilities, has a significant impact on the quality of the associated PWM matrix.

**Results:**

Consequently, we proposed to use receiver operator characteristics curves and the 10-fold cross-validation to learn best weights using experimentally verified data from TRANSFAC database. We applied our method to data available for various TFs. We verified the efficiency of detecting TF binding sites by the 3DTF matrices improved with our technique using experimental data from the TRANSFAC database. The comparison showed a significant similarity and comparable performance between the improved and the experimental matrices (TRANSFAC). Improved 3DTF matrices achieved significantly higher AUC values than the original 3DTF matrices (at least by 0.1) and, at the same time, detected notably more experimentally verified TFBSs.

**Conclusions:**

The resulting new improved PWM matrices for analyzed factors show similarity to TRANSFAC matrices. Matrices had comparable predictive capabilities. Moreover, improved PWMs achieve better results than matrices downloaded from 3DTF server. Presented approach is general and applicable to any energy-based matrices.

EMQIT is available online at http://biosolvers.polsl.pl:3838/emqit.

**Reviewers:**

This article was reviewed by Oliviero Carugo, Marek Kimmel and István Simon.

**Electronic supplementary material:**

The online version of this article (doi:10.1186/s13062-017-0189-y) contains supplementary material, which is available to authorized users.

## Background

DNA-binding site models exist for over 1800 vertebrate TFs and about 3600 known Transcription Factor Binding Sites (TFBSs) in human and over 5000 in mouse. Total number of binding sites in the multicellular genomes could be at least an order of magnitude higher than the number of coding genes [1].

Development of next-generation sequencing methods like ChIP-Seq or ChIP-Chip, covering TF binding over whole genome, remarkably simplifies analysis of gene regulatory regions. Using data coming from such experiments we are able to detect and confirm the exact position and structure of the TFBSs [2, 3]. Bioinformatics approaches are very important for support of experimental identification of protein-DNA interactions.

Binding motifs in DNA are commonly represented by the Position Weight Matrices (PWM) and the Phylogenetic Motif Models (PMM). Experimentally derived PWM models of TFBS profiles are usually deposited in the Jaspar [4] and the TRANSFAC [1] databases. PWM models combined with PWM scanning algorithms score subsequences in the DNA data with respect to their similarity to the TFBS profile, as coded in the PWM. This simple scheme that is commonly used assumes an additive contribution from each position towards the score.

Recently Alamanova et al. [5] devised a method for creating PWMs of transcription factors using 3D structure based computation of protein-DNA free binding energies. The atomistic detail model of TF-DNA interaction would depend on the knowledge of relative spatial configuration of TF amino acids and DNA bases upon binding and a method to evaluate compatibility and strength of TF-DNA interaction. Increasing although still limited number of high quality crystallographic models of TF-DNA complexes deposited in the PDB database [6] allows for detailed study of binding modes and details of contact interfaces. Some recent works report successful structure based predictions of TF binding sites in DNA. Molecular modeling methods require only the 3D structure of the TF-DNA complex. The binding specificity to given DNA motif can be predicted by many different approaches. Molecular dynamics methods, based on the physical energy functions using different terms (electrostatics, solvent release, hydrogen bonds, atom or protein deformations, etc.), model interactions between DNA and protein [7, 8]. Protein-DNA docking allows to create protein-DNA complex for unbound protein structure [9]. The protein-DNA interaction energy is often evaluated by knowledge-based potential. The main idea behind knowledge-based statistical potentials is to analyze contacts between different residue (or atom) types in the complex, based on information from known native structures which are usually protein-DNA complexes deposited in structural databases like PDB. There exist a large number of statistical potentials with different reference state, distance or protein representation [10–12].

Alamanova et al. successfully applied structure based methodology to create PWM matrices of NF-κB family namely p50p50, p50RelB and p50p65 dimers and other factors: p53, GABP and ER*α*. Homology modeling can be used for TF-DNA complexes for which crystallographic data is not yet available. The original Alamanova et al. approach was implemented as the 3DTF web-server available at http://www.gene-regulation.com/pub/programs/3dtf/index.html [5, 13]. Approaches based on crystallographic models and simulation techniques of TF-DNA complexes are a natural intermediate stage between purely bioinformatics-based models and experimental techniques such as ChIP-Chip or ChIP-Seq.

**Fig. 1:**
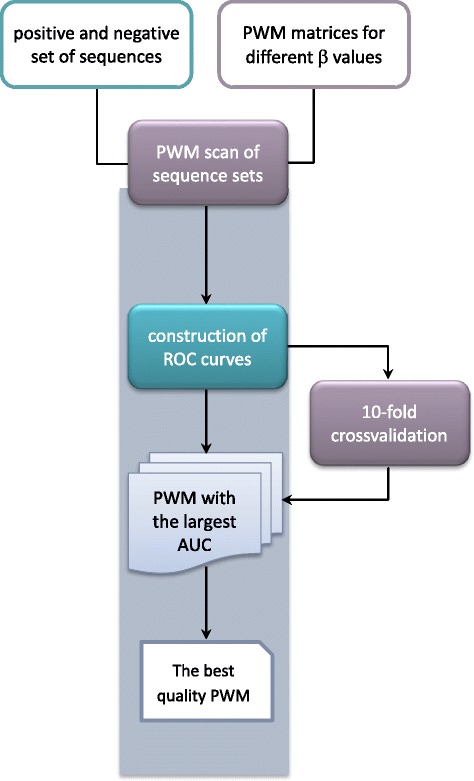
Workflow of PWM matrix improvement procedure

In this work we propose a modification to the Alamanova et al. approach. We observed that tuning of Boltzmann factor weights, used for conversion of calculated energies to nucleotide probabilities, has a significant impact on the quality of the associated PWM matrix [14]. Consequently, we developed EMQIT (Energy Matrix Quality Improvement Tool), a web-server that uses ROC curves and the 10-fold cross-validation to learn the best weights thereby obtaining better predictive models of TFBSs. The method relies of experimentally confirmed TFBSs data form the TRANSFAC database [1]. The general workflow is presented in Fig. 1.

We applied our method to data available for p50p50, p50p65, p53, GABP, HSF1 and ERα. What is very important, our modification significantly improved quality of original 3DTF matrices. The presented approach is general and can be applied to any existing PWM models. The EMQIT web-server is available at http://biosolvers.polsl.pl:3838/emqit.

## Methods

### Dataset

We applied the presented method to data available for the p53 tetramer (PDB entry 2GEQ), ER*α* (1HCQ), GABP (1AWC), HSF1 (3HTS) and for two members of the NF-kB family: p50p50 (1NFK) and p50p65 (1VKX). We improved PWMs computed with 3DTF server. First we downloaded vectors of estimated weights from the 3DTF server [13] consequently they were used to construct PWMs in our machine learning improvement procedure. According to the Gabdoulline et al. [13] 3DTF server is an online implementation of Alamanova et al. method [5], and uses statistical potential developed by Robertson and Varani [15]. However, in authors opinion the implementation must differ in some way from original, as the matrices created with 3DTF, although based on the same PDB structures, differ from those originally published in [5].

We compared PWMs downloaded from 3DTF and improved using our technique with matrices downloaded from the TRANSFAC (2016.1) database and the original 3DTF matrices. The data set created for the p53 consisted of 21 human promoter sequences of 19 genes, which are known to be regulated by p53 transcription factor. In case of remaining TFs namely Erα, GABP, HSF1, p50p50 and p50p65, the data sets contained 26 (23 genes), 12 (11 genes), 26 (13 genes), 19 (15 genes), and 46 (36 genes) fragments of human promoter sequences, respectively*.* We compared improved matrices with all available matrices in the TRANSFAC database for examined TFs. We used the following (the most recent and the highest-quality TRANSFAC matrices available) V$P53_Q3_01 for p53, V$ERALPHA_Q6_02 for Er*α*, V$HSF1_Q6_01 for HSF1, V$P50P50_Q3 for p50p50 and V$P50RELAP65_Q5_01 for p50p65 in the final comparison.

For every considered transcription factor we prepared positive and negative datasets (of equal size - the dimensions of datasets for given TF are given in the previous paragraph) based on information stored in TRANSFAC database. The positive set consisted of short promoter sequences (−1900 to 100 bp from the transcription start site) of human genes known to be regulated by given TF, which included TFBSs used to create all the TRANSFAC PWMs for the TF under study. TFBSs did not have fixed length. The negative set was constructed from randomly selected promoters of human genes from the TRANSFAC database, not regulated by analysed TFs.

### Tuning of Boltzmann factor weights

Original Alamanova et al. method used intermediate energy matrices (generated according to [5]) to create final PWMs. The nucleotide probabilities ${p}_{\alpha}^i$were calculated using Boltzmann formula:

$$ {p}_a^i=\frac{ \exp \left(-\beta \varDelta {G}_a^i\right)}{\sum_{\gamma =1}^4 \exp \left(-\beta \varDelta {G}_{\gamma}^i\right)} $$

where $\varDelta {G}_{\alpha}^i$ is the energy contribution from the particular nucleotide α, γ = [A, T, G, C]. Original Alamanova et al. approach simply assumed *β* = 1/*RT*, where *R* is the universal gas constant and *T* temperature in K. We observed that value of the *β* parameter has significant impact on the quality of associated PWM matrix, measured as number of properly detected experimentally confirmed TFBSs (true positives). In our improvement technique we implement the Boltzmann factor weight (*β)* parameter tuning using Receiver Operating Characteristic (ROC) curves and the 10-fold cross-validation to learn best weights using experimentally confirmed TFBS from TRANSFAC database. For input matrix (e.g downloaded from 3DTF server) subject to improvement, we calculate number of matrices for *β* value ranging from minimum to maximum *β* = 1/*RT* with step equal to 10^−6^ *.* The minimum *β* value was selected independently for each TF and corresponds to a PWM matrix for which nucleotide probabilities at any chosen position are at least equal to 0.6. This step eliminates very unspecific PWMs, for which probabilities of each A, T, G, C nucleotide were equal to 0.25 (what happened for *β* values lower than the selected minimum).

### Construction of ROC curves

We used PWMs created in the previous step to scan the positive and the negative datasets with *Match*™ tool [16]. To construct ROC curves we changed the Matrix Similarity Score (MSS) parameter from 0.60 to 1.00. Consequently, for every PWM matrix, we calculated the number of properly detected transcription factor binding sites in the positive dataset (true positives). We define false positives as TFBSs detected in negative dataset (sequences of genes not regulated by given TF) but also TFBSs detected in positive dataset (sequences of genes known to be regulated by given TF) outside the region annotated as TFBS in TRANSFAC database. This seeming inconsequence is due to the fact that promoter region is considerably longer than true, experimentally confirmed TFBS and may contain motifs which although reported by PWM as TFBSs, located outside region annotated as true TFBS and thus considered false positive.

The ROC curves were constructed in the 10-fold cross validation procedure. We used the classical n-fold cross validation method. The datasets (positive and negative) were randomly split into 10 subsets. A training data was built using nine out of ten subsets, and the remaining one subset was used as the test set. The *k*-fold cross validation is an efficient model evaluation method in cases when independent test data is not available and available dataset does not contain large number of samples. Using the whole dataset in different instances of training and test steps prevents overfitting of the model.

Consequently, a ROC curve was constructed for each PWM matrix. From a set of PWM matrices calculated for different β values, only one PWM matrix, with the largest area under the curve (AUC) value, was selected and used to scan the test set with *Match™* tool. The 10-fold cross validation was repeated ten times. The results (AUC values and β values) were averaged after all ten repeats. Matrix which most frequently appeared as top scoring one (with highest AUC value) was selected and presented as final result.

### The EMQIT tool

Presented method was implemented as a web-server available at http://biosolvers.polsl.pl:3838/emqit/. The tool preforms automated processing of energy matrices, which can be obtained from the 3DTF server. As a result one receives an improved 3DTF matrix represented as nucleotide probabilities and matrix logo. Moreover, EMQIT compares improved PWM with matrices available in the TRANSFAC database and the original 3DTF matrix. The EMQIT application was written in the R/Shiny package [17].

### Example of using the EMQIT tool

We used the EMQIT tool to improve the quality of the energy matrix obtained for the GABP transcription factor (Additional file 1: Figure S2). The energy matrix for the 1AWC complex from the PDB was created using 3DTF server and downloaded as TAB-separated text file. The EMQIT tool performed our improvement procedure using the GABP energy matrix as input and the V$GABP_B TRANSFAC matrix as the reference. The positive set consisted of experimentally verified TFBSs, which were used to construct V$GABP_B TRANSFAC matrix. The EMQIT uses only TFBSs which exact positions can be mapped to the hg38 human reference genome. The GABP positive set included 12 TFBSs in 11 genes. The results of the EMQIT tool are shown under four categories: Summary, Logos, AUC Values and the PWM scan results. The Summary includes the TF name, the improved and the original PWMs, the Logos show motif logos of analyzed matrices and one or more TRANSFAC matrices corresponding to the input TF. The AUC values tab shows two tables: upper table contains information about AUC values obtained for improved and original PWMs, the lower one presents the AUC value for the TRANSFAC matrix or matrices. The PWM scan result stores the information about the number of TFBSs in genes in EMQIT positive set, which were involved in computations. Moreover, it includes comparison of detected TFBSs for the *Match™* MSS thresholds 0.6 and 0.8 for the input and improved matrices. The EMQIT allows downloading of the final improved energy matrix as plain text file. We provided 3DTF P53 energy matrix as an example input. The example matrix is available for download in the main panel.

## Results and discussion

NF-κB family is one of the most important TF families in eukaryotic cells. It takes part in regulation of innate immunity, in carcinogenesis, and interacts with other important families such as p53 and HSF1. Understanding of transcription regulation of NF-κB is important not only for biology but also for medicine. On the other hand, developing novel bioinformatics, physical modeling and evolutionary analysis tools and techniques applicable to NF-κB and its targets, will significantly aid research on other transcription factor families.

The application of the PWM quality improvement method allowed to determine the best *β* parameter value for each considered TF (see Additional file 1: Table S1). Sequence logos of improved PWMs are presented in Additional file 1: Figures S1-S6. Matrix logos were created with the R seqLogo package [18]. The general motifs observed in each matrix logo set were similar. The differences were observed in height of individual letters corresponding to information content (bits). Both NF-κB matrices improved in this study are less specific in their inner part (positions 5 and 6) then matrices obtained from the 3DTF server. Improved matrices were constructed for lower values of β (Additional file 1: Table S1 and Figures S1-S6). There are noticeable differences in our matrix motifs and TRANSFAC PWMs, what can be attributed to different lengths of motifs. To test proposed approach we constructed the ROC curves and computed the AUC values for all matrices (Table 1).

**Table 1:** The AUC values estimated for all analysed matrices

TFs	Improved 3DTF matrix	Original 3DTF matrix	TRANSFAC matrix
The P53 tetramer	0.900	0.534	0.757
ER*α*	0.792	0.605	0.734
GABP	0.906	0.577	0.739
p50p50	0.934	0.683	0.788
p50p65	0.887	0.680	0.767
HSF1	0.908	0.702	0.758

All matrices achieved relatively high values of AUC parameter (min. 0.577 and max. 0.934). The best AUC results were obtained for improved matrices for all cases, improved PWMs had better quality and detected less number of false positive TFBSs than TRANSFAC and original 3DTF matrices (Table 2). An improved p50p50 matrix has the highest AUC value among all considered matrices (0.934). Both improved NF-κB matrices have AUC values more than 0.2 higher than original 3DTF matrices and more than 0.1 higher compared to TRANSFAC matrices. In the other cases the differences in AUC value between improved and original 3DTF matrices were 0.366 for p53, 0.329 for GABP, 0.187 for Erα and 0.206 for HSF1 in favor of the improved PWM matrices. We did not observe a significant changes in AUC values in a group of TRANSFAC matrices, the average AUC value was equal to 0.76. The differences between improved and TRANSFAC matrices were 0.143, 0.167, 0.058 and 0.15 for p53, GABP, Erα and HSF1 respectively, in favor of the improved PWM matrices.

**Table 2:** The number of detected TFBSs by improved and original 3DFT matrices for the MSS 0.6

TFs	Improved 3DTF matrix	Original 3DTF matrix
p53	9	9
ER*α*	9	9
GABP	10	6
p50p50	9	9
p50p65	25	31
HSF1	14	16

We scanned positive data sets created for every TF with *Match*™. The MSS parameter was set to 0.8 (the default value). We compared scan results for improved PWMs and original 3DTF energy matrices (Additional file 1: Figures S7-S12). The best results has been obtained with TRANSFAC matrices (data not shown), which is understandable because all positive data sets were constructed based on these matrices. Improved matrices detected different number of binding sites for all TFs, because of the significant differences in the encoded motifs and lower specificity of these matrices. The improved p50p65 matrix detected 17 out of 46 TFBSs, which represent 37% of the whole positive set for this particular TF. The original 3DTF p50p65 matrix recovered only one less TFBS. The best scan result was obtained for the improved GABP matrix, which found 75% of the positive TFBSs set for the MSS = 0.8 and 83% TFBSs for the MSS = 0.6 (Table 2). For the same TF the original 3DTF matrix detected 42% for the MSS = 0.8 and 50% for the MSS = 0.6. The p53 original 3DTF matrix recovered only 5% TFBSs for the MSS = 0.8, which was the worst result. In comparison, the improved p53 matrix detected 19% TFBSs of the positive set. The remaining improved matrices found 26% TFBSs for p50p50, 35% for HSF1 and 23% for Erα for the MSS = 0.8. Finally improved matrices have the ability to recover extra TFBSs, which were not found by other matrices. We noticed differences between the number of TFBSs reported by original 3DTF matrices and PWMs improved in our procedure for the MSS = 0.8. In all cases improved matrices detected higher number of TFBSs, while for the MSS = 0.6 the number of detected TFBSs were the same for almost all cases.

## Conclusions

The resulting new improved PWM matrices for analyzed factors show similarity to TRANSFAC matrices. Matrices had comparable predictive capabilities. Moreover, improved PWMs achieve better results than matrices downloaded from 3DTF server. Presented approach is general and applicable to any energy-based matrices. Experimentally obtained TFBS motifs are available only for a limited number of TFs what motivates development of distinct computational TFBS modeling techniques. EMQIT allows to create TFBS motif models which performance is comparable to experimental ones. Computational solution is a method of choice when experimental PWM is not available, or its quality is low. For TF-DNA complexes for which crystallographic data is not yet available, protein homology modeling and protein-DNA docking can be used. Our method although based on TRANSFAC database, can be easily modified to include custom sequence data for the TFs of interest.

## Reviewers’ comments

### Reviewer’s report 1

Oliviero Carugo, University of Vienna, Austria.

Reviewer comments:

The manuscript by Karolina Smolinska and Marcin Pacholczyk describes a computational strategy to improve the PWM matrices, originally developed by Alamanova, Stegmaier and Kel (BMC Bioinformatics 11:225), by fine-tuning the values of the Boltzmann exponent. The new matrices are used in a series of predictions, the results of which are compared to those obtained with “old” matrices (TRANSFAC and 3DTF). It seems that there is a considerable improvement of performance. However, detailed results are presented only in Additional file 1: Table S3 (supplementary material), while they should be presented explicitly in the Results and Discussion section.

Author’s response:

*We appreciate Prof. Carugo’s interest in our work and his helpful comments. We moved Tables S2 (now* Table 1)* and S3 (now* Table 2 *) to the Results and Discussion section as suggested.*

Reviewer comment continued:

I also find embarrassing that the data, which were used for this work, are owned by a private company and are not available. I ignore the policy of Biology Direct about this issue but I am personally skeptical about this. There is, however, a serious issue that the Authors should clarify.

Author’s response:

*Although we share Prof. Carugo’s concern about availability of the data, the TRANSFAC is the largest and the highest quality collection of data concerning TFBSs available to date. The TRANSFAC database is commercial product licensed by GeneXplain GmBH and is available to any interested party but the license requires a fee to be paid to the GeneXplain company. The license prohibits the distribution or use of the database outside the institution (laboratory) for which the license was issued.*

Reviewer comment continued:

In lines 128–130 the Authors write “we calculated the number of properly detected transcription factor binding sites in the positive dataset (true positives) and the number of remainder TFBSs detected in positive and negative datasets (false positives).” This sentence is questionable. If the true positives are the TFBSs detected in the positive dataset, the true negative cannot be the TFBS detected in both datasets (positive and negative). Usually a true negative is a TFBSs detected in the negative dataset only. Minor points. In lines 103–104 the Authors write “For every considered transcription factor we prepared positive and negative datasets (of equal size)…” I think that it is important here to write also the (numerical) dimension of these datasets. If they are too small, the value of the conclusions would be questionable. In lines 120–121 the Authors write “we calculate number of matrices for β value ranging from minimum to maximum β = 1/RT”. It is, in my opinion, unclear how the range of β (beta) values were scanned.

Author’s response:

*We made the necessary corrections suggested by the reviewer. The definition of false positives was extended. The sizes of positive and negative datasets are listed in Dataset subsection under Methods section. The description of β value scanning procedure was extended.*

### Reviewer’s report 2

Marek Kimmel, Rice University, USA.

Reviewer comments:

This short paper seems to be an interesting addition to the field of estimation of affinities of transcription factors to binding sites in gene promoters (or elsewhere in the genomes). The idea is to use the Boltzmann formula for binding probabilities, but instead of using the the coefficient beta of the form beta = 1/(RT) (as done in the original paper by Alamanova et al.), to tune it so it optimizes the ROC curves wrt the area under the curve (AUC). This has resulted in quality improvements and made the probability weight matrices (PWM) obtained comparable to those available from TRANSFAC, which are sometimes considered gold standard. In my opinion, the method, although not necessarily optimal, provides good to very good PWMs, but first of all a methodology of constructing PWM, also applicable in other circumstances. My technical comments are summed up in the “Recommendations to Authors”. 1. I am not sure how the methodology is affected by overfitting and how effective the 10-fold cross-validation is. Usually, the data set at researcher’s disposal is split into training and validations sets. The method is trained on the training set and validated based on the validation set. It is a rule it performs worse on the latter. Might the authors at least discuss this issue? 2. I understand that TRANSFAC is used for validation. How different is it from the training date set with respect to the characteristics of the sequences investigated? 3. The Discussion might provide more specific information on other applications of the methodology developed in the manuscript.

Author’s response.

*We appreciate Prof. Kimmel’s interest in our work, his helpful comments and constructive criticism. As every machine learning based workflow also our method’s accuracy suffers from limited number of available data. To ensure the best possible quality of our models we acquired the TRANSFAC license the best quality and largest dataset concerning TFBS available to scientific community. Although the largest source, TRANSFAC still contains very limited number of annotated TFBS. We decided to use as much data as possible for learning purposes. Usually models selected in k-fold cross validation perform better than models simply trained on dataset divided into training and test (or validation) part as one exploits more data in k training sessions* [19]*. Most often k = 10 is selected which represents fair trade-off between model with high bias towards learning set (one training and one testing set) and model with high variance (*e.g. *leave one out, k equal to the number of samples). Ideally one would have additional external validation dataset completely independent from that used in cross-validation procedure to made final evaluation of the model performance in so called “nested cross-validation”* [20] *but given relatively small number of known TFBS we decided to limit our approach to 10-fold cross-validation. This decision is in our view unavoidable trade-off between generality of the model and maximal use of available data. Our goal was also automation of presented procedure (and web server deployment) which always require some simplifying assumptions to be made. Presented approach can be easily modified to include more rigorous model validation as more data will become available.*

### Reviewer’s report 3

István Simon, Institute of Enzymology, Hungarian Academy of Sciences, Budapest, Hungary.

Reviewer comments:

This is a nice and correct work. The authors made some improvement on the accuracy of the original prediction method 3DTF. My only problem, that the original 3DTF server is a hardly used one. The NAR paper reported this server five years ago, were cited 8 times according to the web of science, will the original paper of Alamanova D. et al. published in 2010 received 12 citations. I do not expect that the new EMQIT server will be a popular one. However the procedure used in course of improvement could be useful therefore I recommend the publication of this paper.

Author’s response:

*We thank Prof. Simon for his interest in our work. We hope that our contribution to the field of estimation of affinities of transcription factors to binding sites will cause an increase in usage of computational methods for TFBS modeling.*

## Additional file

Additional file 1: Table S1: The β values selected for final improved matrices. **Figure S1.** The p53 tetramer sequence logos. From top: improved 3DTF matrix, original 3DTF matrix and TRANSFAC matrix. **Figure S2.** The GABP tetramer sequence logos. From the top: the improved 3DTF matrix, the original 3DTF matrix and the V$GABP_B TRANSFAC matrix. **Figure S3.** The Erα sequence logos. From the top: the improved 3DTF matrix, the original 3DTF matrix and the V$ERALPHA_Q6_02 TRANSFAC matrix. **Figure S4.** The p50p50 sequence logos. From the top: the improved 3DTF matrix, the original 3DTF matrix and the V$P50P50_Q3 TRANSFAC matrix. **Figure S5.** The p50p65 sequence logos. From the top: the improved 3DTF matrix, the original 3DTF matrix and the V$P50RELAP65_Q5_01 TRANSFAC matrix. **Figure S6.** The HSF1 sequence logos. From the top: the improved 3DTF matrix, the original 3DTF matrix, and the V$HSF_Q6 V$HSF1_Q6_01 TRANSFAC matrix. **Figure S7.** Results of the improved matrix scan of 21 experimentally confirmed p53 binding sites for the MSS 0.8. **Figure S8.** Results of the improved matrix scan of 12 experimentally confirmed GABP binding sites for the MSS 0.8. **Figure S9.** Results of the improved matrix scan of 26 experimentally confirmed Erα binding sites for the MSS 0.8. **Figure S10.** Results of the improved matrix scan of 19 experimentally confirmed p50p50 binding sites for the MSS 0.8. **Figure S11.** Results of the improved matrix scan of 46 experimentally confirmed p50p65 binding sites for the MSS 0.8. **Figure S12.** Results of the improved matrix scan of 26 experimentally confirmed HSF1 binding sites for the MSS 0.8. (PDF 741 kb)

## Abbreviations

AUC: Area Under the Curve
EMQIT: Energy Matrix Quality Improvement Tool
MSS: Matrix Similarity Score (*Match*™)
PMM: Phylogenetic Motif Models
PWM: Position Weight Matrix
ROC curve: Receiver Operating Characteristic curve
TF: Transcription Factor
TFBS: Transcription Factor Binding Site
