# Supplementary material for: EMQIT: a machine learning approach for energy based PWM matrix quality improvement
Source: Biol Direct. 2017 Aug 1;12:17. doi: 10.1186/s13062-017-0189-y (PMC5539975; doi:10.1186/s13062-017-0189-y)
Supplement: Additional file 1: Table S1: — The β values selected for final improved matrices. Figure S1. The p53 tetramer sequence logos. From top: improved 3DTF matrix, original 3DTF matrix and TRANSFAC matrix. Figure S2. The GABP tetramer sequence logos. From the top: the improved 3DTF matrix, the original 3DTF matrix and the V$GABP_B TRANSFAC matrix. Figure S3. The Erα sequence logos. From the top: the improved 3DTF matrix, the original 3DTF matrix and the V$ERALPHA_Q6_02 TRANSFAC matrix. Figure S4. The p50p50 sequence logos. From the top: the improved 3DTF matrix, the original 3DTF matrix and the V$P50P50_Q3 TRANSFAC matrix. Figure S5. The p50p65 sequence logos. From the top: the improved 3DTF matrix, the original 3DTF matrix and the V$P50RELAP65_Q5_01 TRANSFAC matrix. Figure S6. The HSF1 sequence logos. From the top: the improved 3DTF matrix, the original 3DTF matrix, and the V$HSF_Q6 V$HSF1_Q6_01 TRANSFAC matrix. Figure S7. Results of the improved matrix scan of 21 experimentally confirmed p53 binding sites for the MSS 0.8. Figure S8. Results of the improved matrix scan of 12 experimentally confirmed GABP binding sites for the MSS 0.8. Figure S9. Results of the improved matrix scan of 26 experimentally confirmed Erα binding sites for the MSS 0.8. Figure S10. Results of the improved matrix scan of 19 experimentally confirmed p50p50 binding sites for the MSS 0.8. Figure S11. Results of the improved matrix scan of 46 experimentally confirmed p50p65 binding sites for the MSS 0.8. Figure S12. Results of the improved matrix scan of 26 experimentally confirmed HSF1 binding sites for the MSS 0.8. (PDF 741 kb) [file 13062_2017_189_MOESM1_ESM.pdf]

Supplementary Data for:

EMQIT: A machine learning approach for energy based PWM matrix quality improvement

Karolina Smolinska[1], Marcin Pacholczyk[1*]

[1] Institute of Automatic Control, Silesian University of Technology, Akademicka 16, 44-100 Gliwice, Poland

* To whom correspondence should be addressed. Tel: +48322371086; Fax:+48322372127; Email: marcin.pacholczyk@polsl.pl

Table S1. The β values selected for final improved matrices

TF	Improved matrix
P53	2.33E-04
ERα	3.63E-04
GABP	7.2E-05
p50p50	8.5E-05
p50p65	6.4E-05
HSF1	2.67E-04

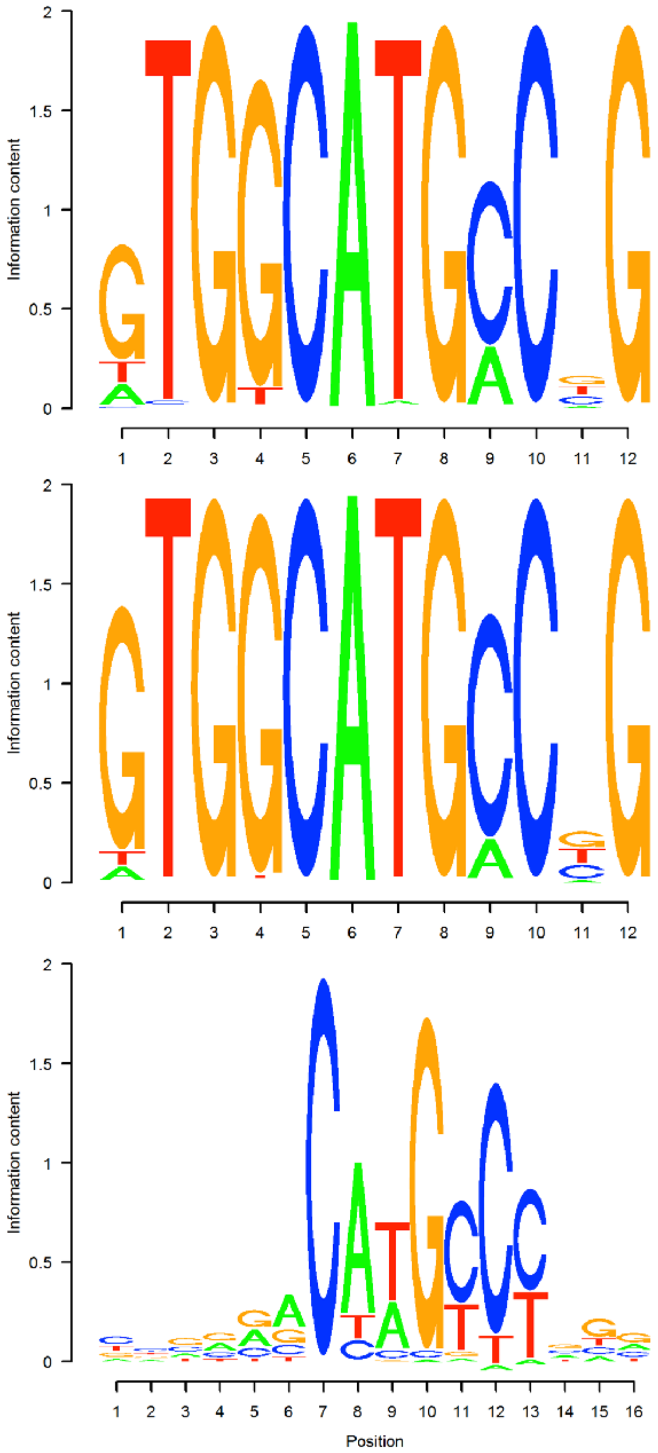

Figure S1. The p53 tetramer sequence logos. From top: improved 3DTF matrix, original 3DTF matrix and TRANSFAC matrix

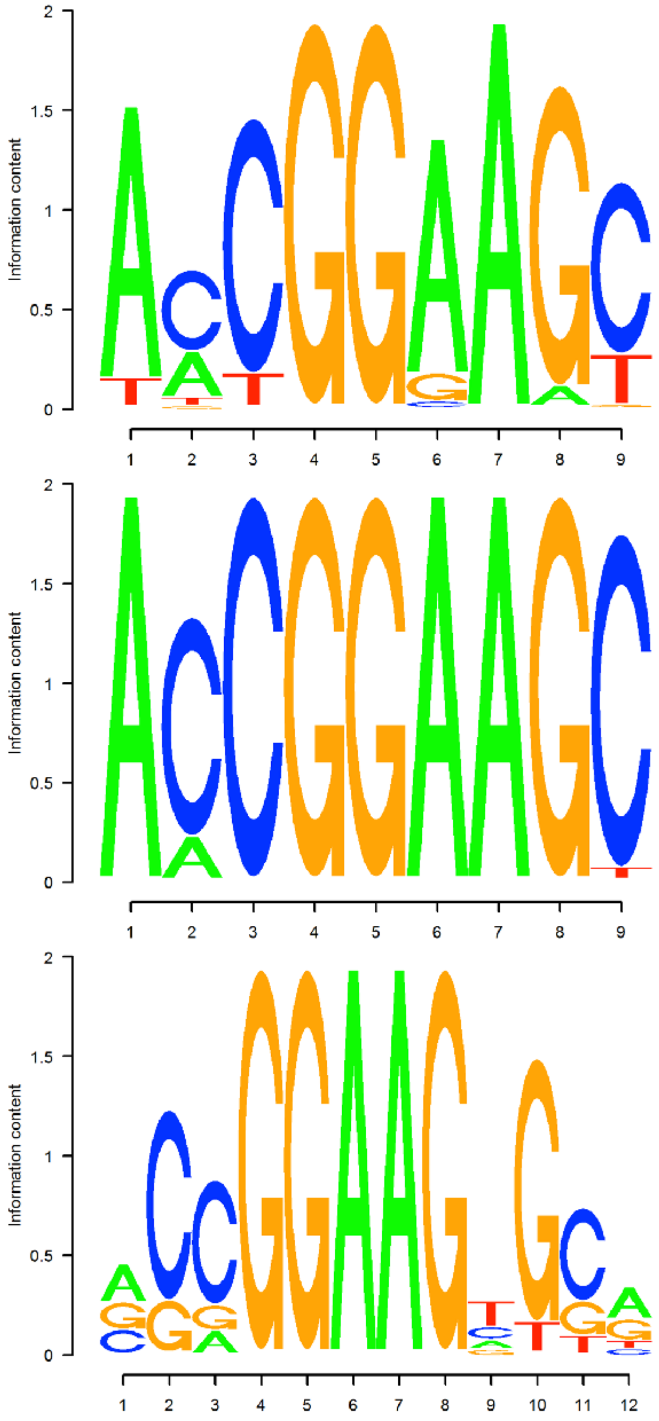

Figure S2. The GABP tetramer sequence logos. From the top: the improved 3DTF matrix, the original 3DTF matrix and the V$GABP_B TRANSFAC matrix

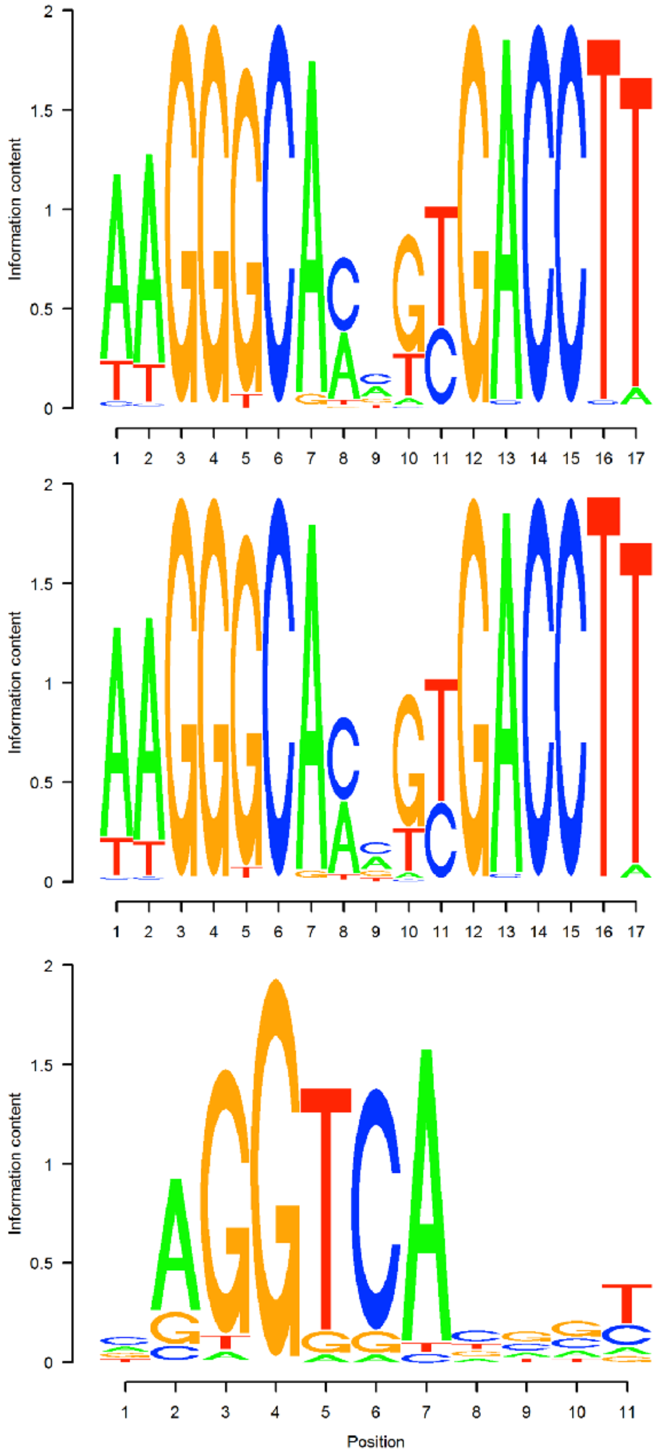

Figure S3. The Erα sequence logos. From the top: the improved 3DTF matrix, the original 3DTF matrix and the V$ERALPHA_Q6_02 TRANSFAC matrix

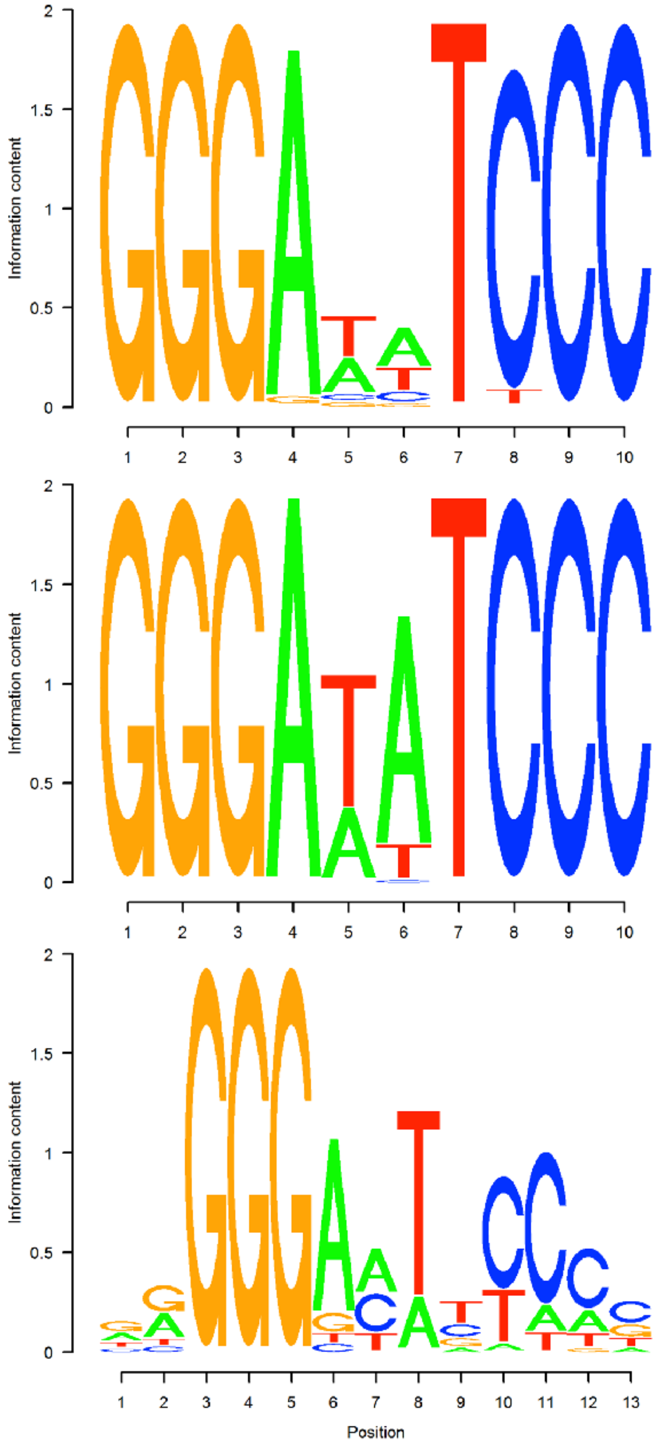

Figure S4. The p50p50 sequence logos. From the top: the improved 3DTF matrix, the original 3DTF matrix and the V$P50P50_Q3 TRANSFAC matrix

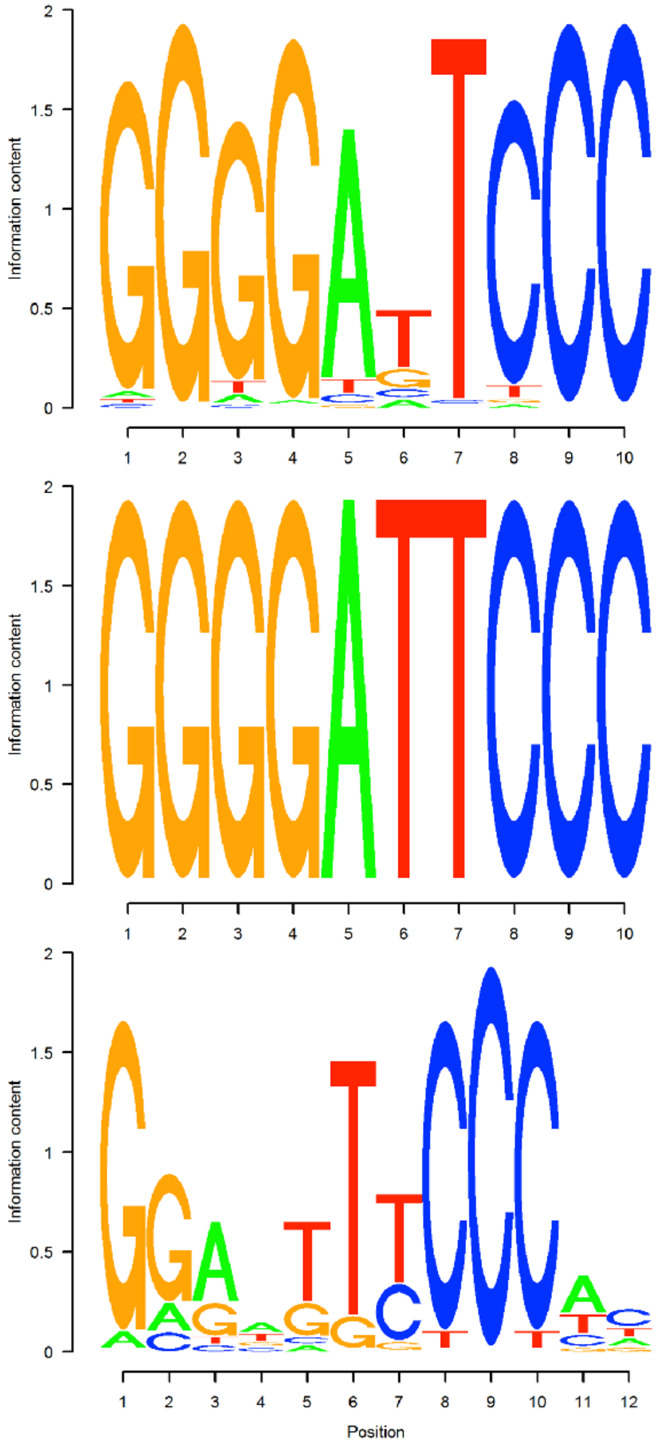

Figure S5. The p50p65 sequence logos. From the top: the improved 3DTF matrix, the original 3DTF matrix and the V$P50RELAP65_Q5_01 TRANSFAC matrix

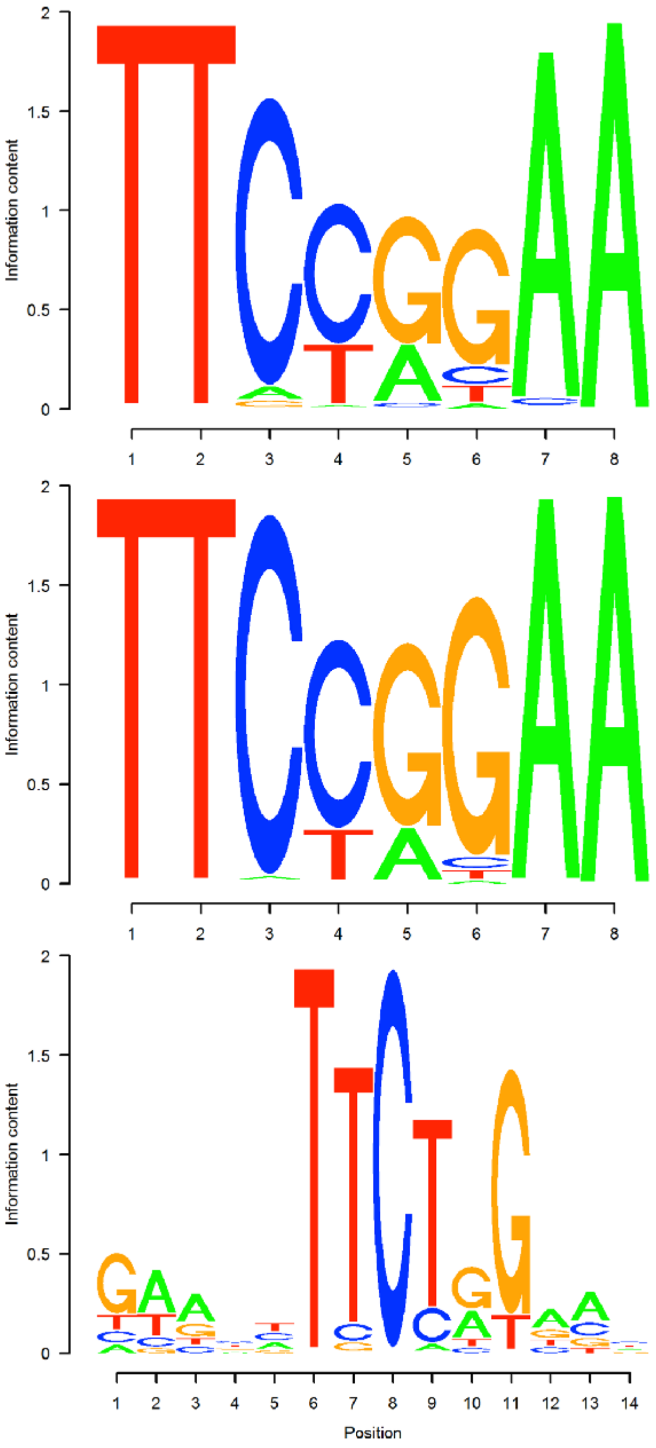

Figure S6. The HSF1 sequence logos. From the top: the improved 3DTF matrix, the original 3DTF matrix, and the V$HSF_Q6 V$HSF1_Q6_01 TRANSFAC matrix

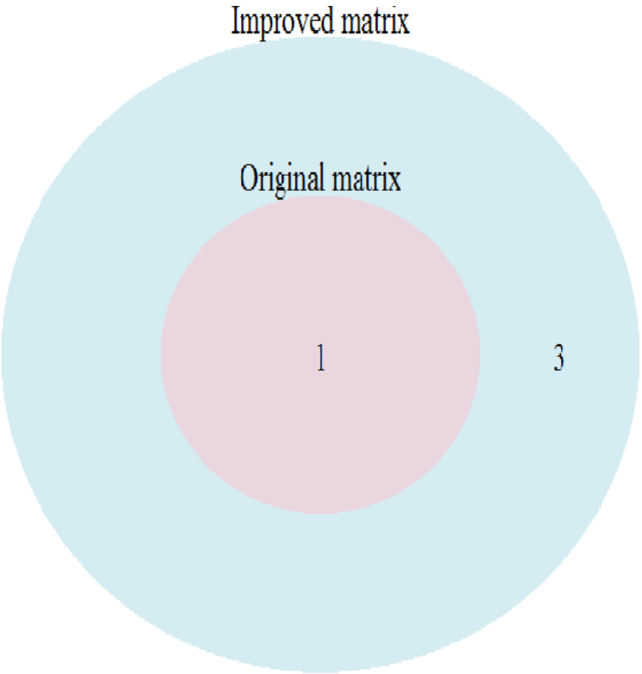

Figure S7. Results of the improved matrix scan of 21 experimentally confirmed p53 binding sites for the MSS 0.8

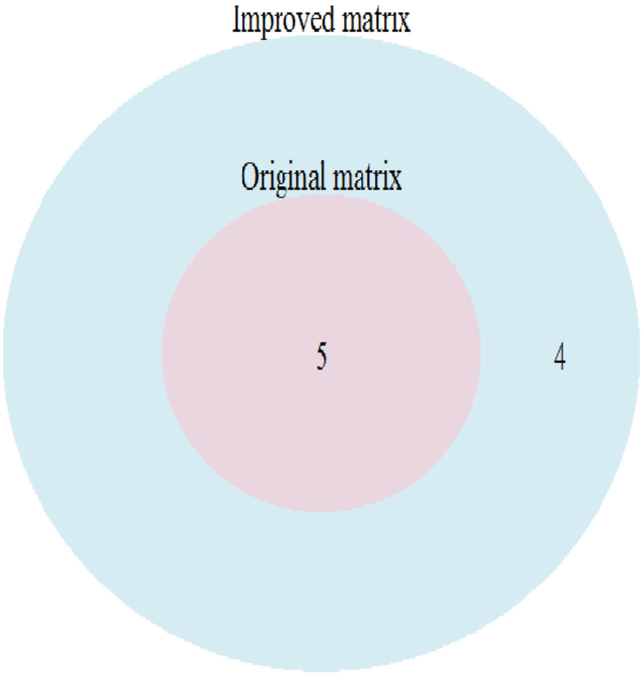

Figure S8. Results of the improved matrix scan of 12 experimentally confirmed GABP binding sites for the MSS 0.8

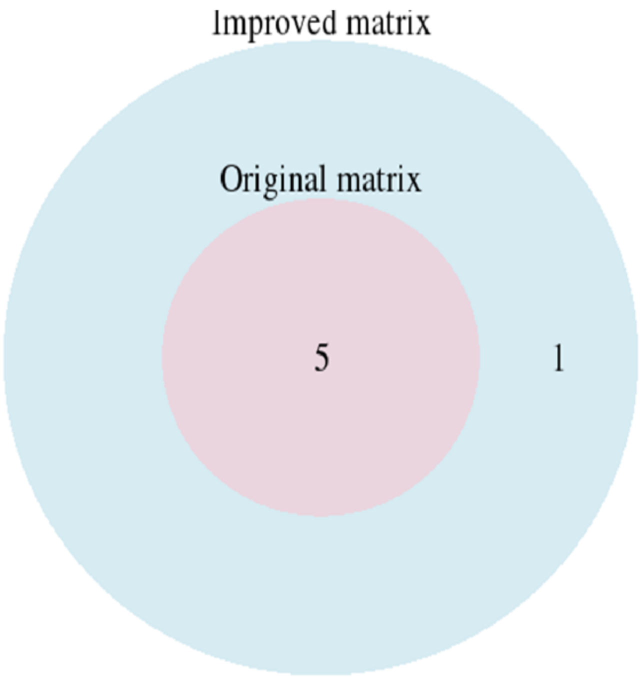

Figure S9. Results of the improved matrix scan of 26 experimentally confirmed Erα binding sites for the MSS 0.8

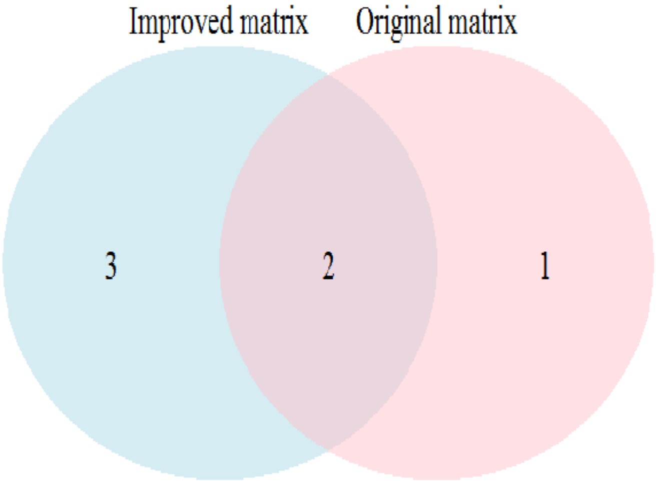

Figure S10. Results of the improved matrix scan of 19 experimentally confirmed p50p50 binding sites for the MSS 0.8

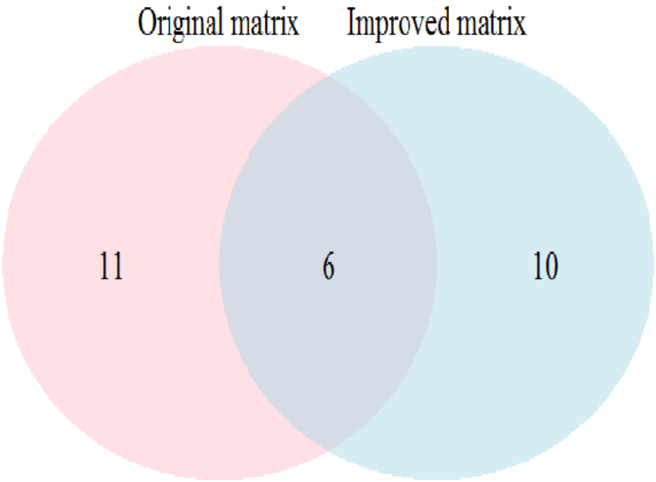

Figure S11. Results of the improved matrix scan of 46 experimentally confirmed p50p65 binding sites for the MSS 0.8

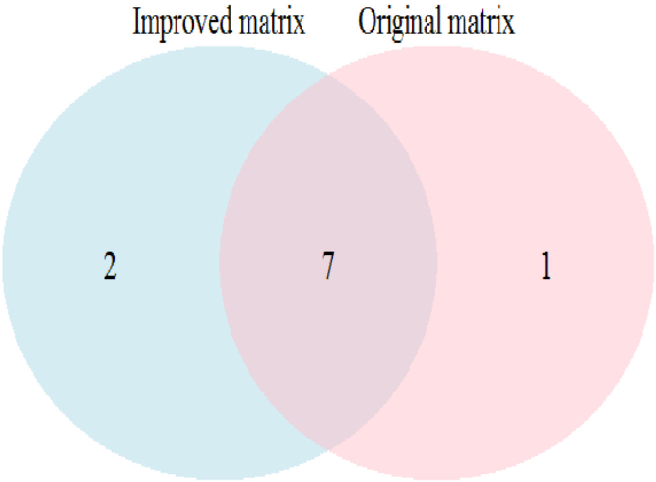

Figure S12. Results of the improved matrix scan of 26 experimentally confirmed HSF1 binding sites for the MSS 0.8
